# Functional Connectivity and Feature Fusion Enhance Multiclass Motor-Imagery Brain–Computer Interface Performance

**DOI:** 10.3390/s23177520

**Published:** 2023-08-30

**Authors:** Ilaria Siviero, Gloria Menegaz, Silvia Francesca Storti

**Affiliations:** 1 Department of Computer Science, University of Verona, Strada Le Grazie 15, 37134 Verona, Italy; ilaria.siviero@univr.it; 2 Department of Engineering for Innovation Medicine, University of Verona, Strada Le Grazie 15, 37134 Verona, Italy; gloria.menegaz@univr.it

**Keywords:** functional brain connectivity, motor-imagery brain–computer interface, translation-invariant features, scattering convolution network, feature fusion, multiclass classification

## Abstract

(1) Background: in the field of motor-imagery brain–computer interfaces (MI-BCIs), obtaining discriminative features among multiple MI tasks poses a significant challenge. Typically, features are extracted from single electroencephalography (EEG) channels, neglecting their interconnections, which leads to limited results. To address this limitation, there has been growing interest in leveraging functional brain connectivity (FC) as a feature in MI-BCIs. However, the high inter- and intra-subject variability has so far limited its effectiveness in this domain. (2) Methods: we propose a novel signal processing framework that addresses this challenge. We extracted translation-invariant features (TIFs) obtained from a scattering convolution network (SCN) and brain connectivity features (BCFs). Through a feature fusion approach, we combined features extracted from selected channels and functional connectivity features, capitalizing on the strength of each component. Moreover, we employed a multiclass support vector machine (SVM) model to classify the extracted features. (3) Results: using a public dataset (IIa of the BCI Competition IV), we demonstrated that the feature fusion approach outperformed existing state-of-the-art methods. Notably, we found that the best results were achieved by merging TIFs with BCFs, rather than considering TIFs alone. (4) Conclusions: our proposed framework could be the key for improving the performance of a multiclass MI-BCI system.

## 1. Introduction

A motor-imagery brain–computer interface (MI-BCI) is a system that can translate human brain activity into real-time control signals or computational commands [1]. Using the power of mental imagination, MI-BCI systems allow users to interact with the external world without any physical movement [2,3].

The widely used modality to capture brain activity for MI-BCI systems is electroencephalography (EEG), known for its non-invasive nature, high temporal resolution, and cost-effectiveness [4,5]. During MI tasks, the μ (8–12 Hz) rhythm in the contralateral motor-sensory area decreases as users imagine, attempt, or prepare for a specific movement. This phenomenon, known as event-related desynchronization (ERD), is followed by event-related synchronization (ERS) in the μ (8–12 Hz) and in β (13-30 Hz) frequency bands after the completion of the movement or its imagination [4].

MI-BCI systems have a broad spectrum of applications in medical (e.g., motor rehabilitation) and non-medical fields (e.g., smart environments, games, and industry) [5], but they still face challenges in real-world scenarios [6,7]. Although promising signal processing frameworks have been proposed in the literature, distinguishing more than two MI tasks remains a major challenge. Indeed, MI tasks frequently involve multiple interconnected cortical regions, and ERD/ERS can occur in different parts of the sensorimotor cortex, at different frequency bands, and at different times intervals [6,8]. Consequently, there is a compelling need to explore novel approaches that can offer more comprehensive insight into the underlying neuronal processes. One such approach involves brain connectivity analysis, which has proved to be beneficial in enhancing the performance of MI-BCI systems [9,10].

Brain connectivity is a powerful tool for analyzing connections from brain recordings and quantitatively assessing the interaction between neurobiological systems [11,12]. These neural systems interact with local or distant regions during cognitive, motor, and MI tasks, exhibiting unilateral or bilateral synchronization.

The two main measures used for functional coupling analysis in EEG signals are functional connectivity (FC) and effective connectivity (EC) [6,13]. FC explores only the statistical dependency and temporal correlation between two time series and can be estimated using linear, non-linear, and information-based approaches. Some examples of FC are Pearson correlation, coherence, the phase lag value (PLV), and the phase lag index (PLI). Among these techniques, phase correlation (PC) has demonstrated robustness in classifying epileptic seizures via EEG signals, allowing the capture of relative changes between two brain signals [14].

On the other hand, EC reflects the causal interaction between brain activities, specifically the direct or indirect influence and amplitude exerted by one neural system on another [15,16]. These approaches, along with novel strategies like graph-theory-based methods, have been employed to investigate the network properties of brain connectivity and its dynamics [17,18,19,20,21]. Despite their extensive use in neuroscience [20], brain connectivity measures have only recently gained attention in the MI-BCI field [22]. Thus, investigating brain connectivity offers valuable insights into the statistical dependencies between well-organized brain regions, improving the discrimination power among MI tasks [6].

The typical MI-BCI signal processing pipelines involve preprocessing, feature extraction, and classification [1]. The preprocessing step reduces noise and removes artifacts from the EEG signals, while feature extraction plays a crucial role in transforming the processed brain signals into features reflecting the user’s intent. Finally, a classification model is required to distinguish brain patterns among different MI tasks. A widely adopted machine learning (ML) classifier is the support vector machine (SVM), which provides high classification accuracy with low computational complexity [5,22]. In addition, there exist other classification models that could be considered, such as linear discriminant analysis (LDA), k-nearest neighbor (k-NN), the random forest (RF) classifier, and logistic regression [5,22]. A recent analysis of the existing literature on brain connectivity in MI-BCIs revealed that SVMs were exploited in approximately 48% of the considered works; LDA in around 27%; and other classifiers, such as RF, in only around 2% [22]. However, attempts have been made using deep learning (DL) methods, such as convolutional neural network (CNN) and recurrent neural network (RNN) approaches, but these methods are more complex due to their learning processes and require the availability of a large amount of data from multichannel EEG. Therefore, traditional ML models are preferred due to their ability to achieve high classification accuracy with lower computational complexity [23].

The combination of these steps is decisive in developing robust and accurate MI-BCI systems. Among them, the most crucial step is the feature extraction phase, where current methodologies present some limitations, especially in multiclass scenarios [24]. In the literature, this step is typically performed in the spatial, frequency, and/or time domain by analyzing the information coming from the local brain region. Recently, scattering convolution networks (SCNs), also known as wavelet scattering networks, have proven effective for extracting discriminative features from EEG signals recorded during a cognitive task [25] and have shown promising results in the MI-BCI domain [26,27]. SCNs use wavelet filters to extract translation-invariant features (TIFs), making them robust to shifts in input data. Moreover, they provide interpretable features that facilitate EEG signal analysis [28]. The fixed filter weights of SCNs eliminate the need for a learning process, which significantly reduces computational complexity and is effective with a limited amount of data [29].

Channel selection is another important aspect affecting MI-BCI performance. Typically, one or multiple EEG channels are selected based on a priori neurophysiological knowledge and are used independently to decode the brain activity. Channels commonly considered in MI tasks include $C_{3}$ and $C_{4}$, which are associated with hand movement imagination, and $C_{z}$, which is associated with foot movement imagination [30,31,32]. Instead of using channels selected based on a priori knowledge, some researchers have proposed channel-selection approaches that aimed to find the most effective features from EEG recordings. For example, in [33], the authors proposed a novel approach that used the minimum redundancy maximum relevance paradigm to reduce the number of channels, achieving a 53% reduction rate. This result highlights the importance of considering channel-selection strategies in order to improve the performance of MI-BCI systems.

All the above-mentioned methods are based on single or few EEG channels, and the information conveyed by them may not be sufficient to identify the user’s intention, especially when the problem to be solved is multiclass and the brain areas involved are very close [34,35]. To address these issues, the exploitation of brain connectivity as a feature extraction technique has recently gained popularity in the MI-BCI community, particularly in binary classification [6,22]. Examples of FC metrics used for classification include the PLV, the PLI, and the weighted phase lag index (wPLI) [9,24,36]. In addition to FC analysis, some studies have also explored EC analysis. Notably, Liang et al. proved that there is an information flow between the two hemispheres during the imagination of right- and left-hand movements. This was achieved using the partial directed coherence (PDC), an EC measure designed to estimate the intensity of information flow [37]. Another study proposed a novel EC measure based on Renyi’s transfer entropy, which yielded discriminant spatiotemporal patterns for distinguishing between right- and left-hand MI tasks [38].

Brain connectivity analysis remains relatively unexplored in the case of the multiclass MI-BCI problem [9,22]. Only in recent years, Uribe et al. used the correntropy, Pearson correlation, and Spearman correlation as FC features, followed by a graph brain network analysis [23]. Some studies aimed to compare different brain connectivity metrics, but classification performances were still limited when compared to other state-of-the-art (SOA) methods [23,39,40]. Rodrigues et al. highlighted that brain connectivity results may be sensitive to EEG characteristics such as the particular session, the day or week, the electrode positions, and impedance [39]. Therefore, in addition to the experimental conditions, several factors may influence MI-BCI performance due to the variability in EEG recordings. This intra-subject variability is related to changes in neural processing over time, the non-stationarity of EEG signals, and numerous neurophysiological mechanisms [41]. It is important to note that all these factors cause variations in EEG-based feature distribution, known as the covariate shift, resulting in a decrease in performance [34,42].

Furthermore, a highly promising approach for further improving classification results in MI-BCI applications is the feature fusion technique [43]. By combining different types of features extracted from EEG signals, this approach creates a comprehensive feature vector that captures a broader range of information essential for MI tasks. This significantly enhances the discriminative power of the classification model, leading to more accurate and reliable results. The concept of feature fusion has recently been adopted in the MI-BCI field [36,44], demonstrating successful classification results in not only this domain but other contexts as well [45]. An example comes from Wang et al., who introduced a novel MI-BCI framework by fusing local and global features, extracted using the PLV and the one-versus-the-rest filter-bank common spatial pattern (OVR-FBCSP) method [36]. Using a dataset shared by the Anhui University containing three MI tasks, they demonstrated how the integration of brain connectivity features with channel-based features could significantly enhance the performance of the MI-BCI system. Another feature fusion approach was proposed by Ai et al., which fused features of the brain networks with features in the frequency and spatial domains extracted by the common spatial pattern (CSP) and local characteristic scale decomposition (LCD) algorithms. They demonstrated that the proposed method was robust in discriminating brain waves during MI tasks.

The aim of this study is to present a novel feature fusion framework for enhancing the classification of multiclass MI tasks. The proposed framework’s architecture combines features invariant to translation derived from the SCN with traditional FC features. This combination of distinct features can provide a holistic view of brain activity, obtaining interpretable features and capturing the interconnections among distinct brain regions. The impact of the proposed pipeline on the MI-BCI system performance is evaluated using a multiclass SVM as the classifier. In addition, a new evaluation strategy is presented to deal with intra-subject variability among different EEG sessions. Our proposed method demonstrates its efficacy in improving the performance of an MI-based BCI system, with potential benefits for both individuals with disabilities and those who are neurologically healthy.

## 2. Materials and Methods

**Figure 1:**
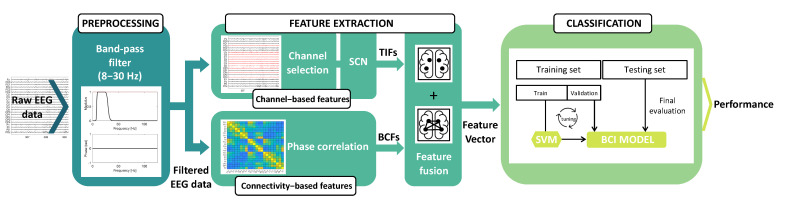
Overview of the proposed MI-BCI signal processing framework. Preprocessing: the raw EEG data are filtered using a band-pass filter (cutoff frequencies: 8–30 Hz). Feature extraction: the channel-based features are extracted using the SCN on a subset of EEG channels, as shown in the upper part of the figure. The lower part of the figure illustrates the extraction of BCFs computed by applying PC. Classification: the merged TIFs and BCFs are classified by a multiclass SVM classifier.

The proposed MI-BCI signal processing pipeline, which consists of preprocessing, feature extraction, and classification steps, is outlined in Figure 1. In Section 2.1, we provide a description of the dataset and the preprocessing step. Section 2.2 and Section 2.3 delve into feature extraction, feature selection, and channel selection. Lastly, in Section 2.4 and Section 2.5, we present the classification strategy and the performance assessment, respectively. The dataset used in this analysis was dataset IIa from the BCI Competition IV [46].

### 2.1. Dataset Description and Preprocessing

**Figure 2:**
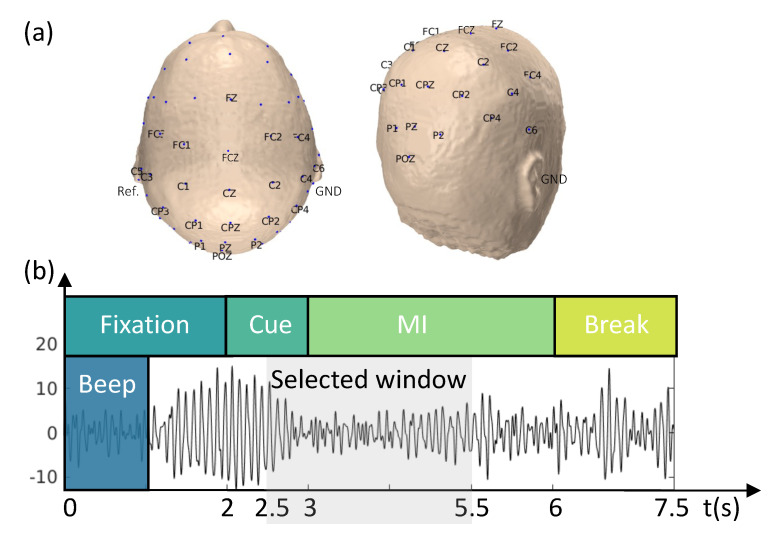
Schematicrepresentation of the experimental paradigm and EEG montage. (**a**) The 22-channel EEG montage used in the study. The EEG was recorded monopolarly, with the left mastoid serving as a reference and the right mastoid as the ground. (**b**) The task was divided into four stages: (i) fixation (2 s), (ii) cue-based signal (1.25 s), (iii) MI task (3 s), and (iv) a short break (1.5 s).

The proposed methodology was evaluated using a publicly available EEG dataset from the BCI Competition IV, dataset IIa, which contains data from 9 subjects [46]. The cue-based BCI paradigm included four distinct MI tasks: the imagination of the movement of the left hand (LH), right hand (RH), both feet (F), and tongue (T). For each subject, two EEG sessions were recorded on different days using a cap with 22 EEG electrodes plus 3 monopolar electrooculography (EOG) channels (Figure 2a). Signals were recorded monopolarly, with the left mastoid serving as a reference and the right mastoid as the ground. Each session consisted of six runs, separated by brief breaks, with 288 trials (72 cue-based trials for each of the four classes).

Each trial was 7.5 s long, during which participants were not provided with any feedback. The trial began with a 2 s fixation period, followed by the appearance of a cue-based signal on the monitor for 1.25 s. The participants then performed one of four randomly assigned MI tasks. The paradigm is illustrated in Figure 2b. The EEG signals were sampled at a frequency of 250 Hz and band-pass-filtered between 0.5 and 100 Hz. A 50 Hz notch filter was then applied to reduce line noise.

The analysis focused on the period between 2.5 and 5 s, during which the ERD/ERS was more prominent (as shown in Figure 2b). A zero-phase Butterworth filter was applied between 8 and 30 Hz to preserve both the α (8–12 Hz) and β (13–30 Hz) bands [37,44,47,48]. To achieve a zero-phase filter, the filtering process was repeated twice, once in the forward and once in the reverse direction on the signal time history. A baseline correction was applied to reduce the effect of temporal drifts, and the EEG signals were re-referenced to the CAR. Trials containing artifacts, as identified in the dataset [46], were excluded from the analysis.

### 2.2. Brain Connectivity Features (BCFs)

To extract BCFs and detect changes between two signals, three different FC metrics were used: Pearson correlation [11], the PLI [49], and PC [14]. The use of Pearson correlation and the PLI in the MI-BCI field has been previously reported [6,24,39], especially in the case of binary classification tasks. On the other hand, the PC metric has been applied for classifying epileptic seizures [14].

Let us define the PLI and PC by introducing the formulation of the instantaneous phase ϕ(t). Given a time series x(t), the analytical signal z(t) is complex-valued, where $x_{H}\left(t\right)$ represents the Hilbert transform, A(t) denotes the instantaneous amplitude, and ϕ(t) represents the instantaneous phase. These are defined as follows:

(1) $$z\left(t\right)=x\left(t\right)+ix_{H}\left(t\right)=A\left(t\right)e^{i\phi (t)}$$

In particular, the Hilbert transform of x(t) is calculated as follows:

(2) $$x_{H}\left(t\right)=\frac{1}{\pi }p.v.\int_{-x}^{\infty }\frac{x(\tau )}{t-\tau }d\tau$$

in which p.v. represents the Cauchy principal value of the integral [49].

Finally, the instantaneous phase ϕ(t) is defined as:

(3) $$\phi \left(t\right)=arctan\frac{x_{H}\left(t\right)}{x(t)}$$

The instantaneous amplitude A(t) is defined as:

(4) $$A\left(t\right)=\sqrt{{\left[x_{H}\left(t\right)\right]}^{2}+{\left[x\left(t\right)\right]}^{2}}$$

The PLI is defined as follows:

(5) $$PLI=|\frac{1}{N}\sum_{t=1}^{N}sign\left[\Delta \phi \left(t\right)\right]|$$

where Δϕ(t) is the phase difference between two signals.

The PC is calculated as follows:

(6) $$r\left(\phi_{i},\phi_{j}\right)=\frac{cov(\phi_{i},\phi_{j})}{\sqrt{var\left(\phi_{i}\right)var\left(\phi_{j}\right)}}$$

with $1\leq i\leq n_{c}$ and $1\leq j\leq n_{c}$, where $n_{c}$ is the total number of EEG channels. BCFs were calculated for each MI epoch, resulting in symmetric adjacency matrices. This resulted in a total of $n_{k}\left(n_{c}*n_{c}\right)$ features, where $n_{k}$ is the number of epochs. Since the matrix is symmetric, each matrix related to one epoch can be transformed into a vector of $n_{c}\left(n_{c}-1\right)/2$ values. For example, when using an EEG system with 22 electrodes, this results in a total of $n_{k}*231$ BCFs for each class. We used all EEG channels to calculate the adjacency matrix. It is worth noting that PC and Pearson correlation range between 1 and −1, while the PLI varies between 0 and 1.

### 2.3. Translation-Invariant Features (TIFs) and Channel Selection

Translation-invariant features are extracted by scattering wavelets [28]. The wavelet transform is a powerful tool for extracting features that are well-localized in both time and frequency through convolution operations with translated and dilated wavelets ψ(u). However, this method is suboptimal for classification, as convolutions are translation-*covariant*, rather than translation-*invariant*. To obtain TIFs, the scales must be separated, and non-linearity must be applied [29]. The SCN is a method that builds non-linear invariants from wavelet coefficients by introducing modulus and average pooling functions. Higher-order coefficients are obtained by iterating wavelet transforms and modulus operators. The structure of an SCN is similar to that of a traditional deep convolutional neural network (CNN), where convolutional filters are aligned with wavelet filters, the rectified linear unit (ReLU) corresponds to the modulus operator, and pooling is accomplished through low-pass filtering. The advantages offered by SCNs include the use of fixed filter weights instead of learning the filters from the data, leading to a reduced computational complexity. Furthermore, the extracted features are invariant up to a specified scale, and the scattering transform retains the energy of the signal [28]. In this work, we fixed the invariance scale to 2 s, as this provides the best classification results according to previous research [26]. To extract TIFs, we specifically chose a subset of channels consisting of central, fronto-central, and centro-parietal channels instead of using all 22 EEG channels [36]. This subset included half of the total number of channels (i.e., $n_{c}$).

### 2.4. Feature Selection and Classification

The final feature vector was created by combining two different types of features, referred to as TIFs and BCFs, through a process called feature fusion [43]. If *A* and *B* are the two feature spaces of TIFs and BCFs, respectively, and if α∈A and β∈B are the two feature vectors, the feature fusion is defined as γ=αβ. For example, if α is *n*-dimensional and β is *m*-dimensional, the final feature vector is (n+m)-dimensional.

The feature selection was performed using the Fisher’s score in its multiclass version [24]. This method assigns a score to each feature vector based on its ability to differentiate between classes in the dataset. The final set of features includes the features with the highest scores. The Fisher’s score of the *j*-th feature is defined as:

(7) $$F\left(X^{j}\right)=\frac{\sum_{k=1}^{4}n_{k}{\left(\mu_{k}^{\left(j\right)}-\mu^{\left(j\right)}\right)}^{2}}{\sum_{k=1}^{4}n_{k}{\left(\sigma_{k}^{\left(j\right)}\right)}^{2}}$$

where $\mu_{k}^{\left(j\right)}$ is the mean and $\sigma_{k}^{\left(j\right)}$ is the standard deviation of the *j*-th feature in the *k*-th class; $n_{k}$ is the cardinality of the *k*-th class; $j=1,2,..,N_{f}$, with $N_{f}$ representing the total number of features; and $\mu^{\left(j\right)}$ denotes the mean of the *j*-th feature on the whole dataset. The final set of features, *S*, with the highest value of $F(X^{j})$ was selected for further analysis.

For classification, we used an SVM model with a radial basis function (RBF) kernel, a well-established classifier in the BCI field [5]. SVM models have gained popularity due to their ability to provide interpretable results with relatively small amounts of data [22]. The hyperparameters of the RBF kernel, *C* and γ, were tuned using a grid search on the training set. Since SVMs are designed for binary classification problems, we employed the one-versus-the-rest (OVR) approach to solve the multiclass problem [50,51].

### 2.5. Performance Assessment

**Figure 3:**
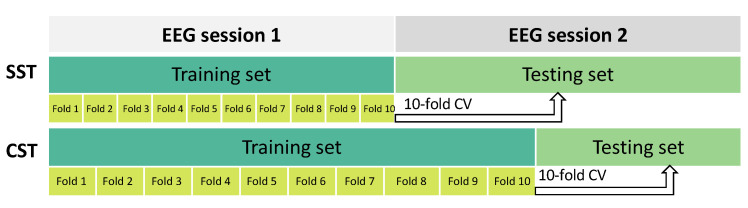
The dataset was divided using two different configurations to evaluate the performance of the MI-BCI system. In the upper part of the figure, the *session-to-session transfer* (SST) setup is shown. Here, the first EEG recording session was used as the training set and the second EEG session as the testing set. This partitioning setup simulated a scenario wherein knowledge learned from one recording session is applied to a different EEG session for evaluation. In the lower part of the figure, the *calibration-session transfer* (CST) division is depicted. In this setup, the first session and a portion (40%) of the second one were used as the training set. The remaining portion (60%) of the second session was used as the testing set. This partition strategy simulated a scenario wherein part of a recording session is used for calibration and the remaining part is used for testing the system performance. In both cases, a 10-fold CV was performed on the training set.

In this study, two evaluation strategies were used to assess the performance of the SVM classification model with the selected features, as shown in Figure 3:

1. *Session-to-session transfer (SST)*: Session 1 of the EEG recordings was used as the training set and session 2 as the testing set [35,52].
2. *Calibration-session transfer (CST)*: To account for the variability of EEG recordings [42], a *calibration* session was introduced. In this strategy, the training phase was performed using both session 1 of the EEG recordings and a portion of session 2. To achieve this, we split the original test set (i.e., session 2) into two different parts: the first 40% was added to session 1 for training, and the remaining 60% was used for testing. The split was performed in a balanced manner among different classes.

In the training phase, a 10-fold cross-validation (CV) was performed to avoid overfitting. At each step of the CV, the Fisher’s score was computed to select the features.

The performance was evaluated mainly in terms of accuracy (Acc) and Cohen’s kappa value (*k*). The classification accuracy refers to the ratio of correctly predicted target class epochs, calculated as follows:

(8) $$Acc=\frac{\mathrm{TP}+\mathrm{TN}}{\mathrm{TP}+\mathrm{TN}+\mathrm{FP}+\mathrm{FN}}$$

where TP indicates true positive, TN true negative, FP false positive, and FN false negative. The Cohen’s kappa value is a measure of agreement between predicted and true labels. This measure is particularly useful in cases of unbalanced classes. The Cohen’s kappa value ranges between 1 and −1. A Cohen’s kappa value equal to 1 denotes a perfectly correct classification and a value of −1 an erroneous classification, while a value of 0 indicates no correlation between the predicted and true labels. The Cohen’s kappa value is defined as follows:

(9) $$k=\frac{p_{o}-p_{e}}{1-p_{e}}$$

where $p_{o}$ is the observed agreement, and $p_{e}$ is the expected agreement. To further validate the performance of the model, precision (Prec) and recall (Rec) were reported, which were calculated as follows:

(10) $$Prec=\frac{\mathrm{TP}}{\mathrm{TP}+\mathrm{FP}}\;\;Rec=\frac{\mathrm{TP}}{\mathrm{TP}+\mathrm{FN}}$$

To evaluate the impact of FC on the MI-BCI performance, the classification results are reported in two different cases:

- Case 1: the feature vector contained only TIFs extracted with the SCN;
- Case 2: the feature vector included TIFs and BCFs.

Finally, a Wilcoxon signed rank test was performed to test the difference in performance between the two cases for both the SST and CST approach. The results for four-class classification are presented for both the SST and CST scenarios (both case 1 and case 2), and the results for three-class classification are presented for the SST scenario (only case 2).

## 3. Results

The main finding of this work confirmed that incorporating brain connectivity information can improve the performance of a MI-BCI system by merging channel-based features with network ones. In particular, the classification accuracy achieved using the SST approach was found to be higher when using both TIFs and PC (case 2) compared to using only TIFs (case 1). The results of the SST approach are presented in Table 1 and Table 2, which show that high values of validation accuracy were achieved for both cases 1 and 2. The average validation accuracy across subjects was 83.76% (case 1) and 84.88% (case 2), with the highest validation accuracy being 95.27% for subject 9 (case 2). The average test accuracy across subjects was 47.82% and 56.10% in case 1 and case 2, respectively, with case 2 achieving an accuracy 8.28% greater than case 1. The Wilcoxon signed rank test revealed a significant difference in performance between case 1 and case 2 (p=0.0039) for the SST approach. These findings were further confirmed by additional evaluation metrics. Both the average validation and test values across subjects were higher in case 2. In particular, for case 1, the average Cohen’s kappa test value was 0.30, the average test precision was 0.47, and the average test recall was 0.48. For case 2, these values improved to an average Cohen’s kappa test value of 0.41, an average test precision of 0.57, and an average test recall of 0.56.

In Table 3, the results obtained by employing different BCFs are reported for the SST evaluation approach. Both the validation and test accuracy using TIFs fused with PC, Pearson correlation, and the phase lag index, respectively, are presented. As shown, TIFs merged with the PC measure outperformed the other two metrics, achieving an average test accuracy of 56.10% compared to 51.31% and 49.05%. Consequently, we chose to use TIFs fused with PC for our subsequent analysis.

As mentioned in Section 2.5, these results encouraged us to investigate the CST evaluation approach, since the EEG (and thus FC) seemed to be strongly influenced by the EEG session. The results obtained by CST are summarized in Table 4. We achieved an average test accuracy across subjects of 63.25% in case 1 and 71.67% in case 2, with an improvement of 8.42%. The best test classification accuracy was 88.82% for subject 3 and 91.45% for subject 9 for the CST approach (in case 2). Again, the testing results were highly subject-dependent, but the standard deviation (SD) was lower in comparison to the previous case (in case 2, the SD was equal to 18.54 and 13.89 for SST and CST, respectively). The Wilcoxon signed rank test indicated a significant difference in performance between case 1 and case 2 (p=0.0156) for the CST approach.

Moreover, the differences between the average validation and test accuracy were lower for the CST approach, at 12.74% (case 1) and 6.48% (case 2) compared to values of 35.94% (case 1) and 28.78% (case 2) obtained by the SST strategy. Thus, the CST strategy helped to not only reduce overfitting but also improve the classifier’s ability to generalize, as the heterogeneity of the class was better represented. Finally, by comparing the two evaluation strategies, we observed that the average test accuracy across subjects enhanced from 56.10% to 71.67%. Of note, the difference between the average validation and test accuracy achieved by SST and CST (in case 2) was 28.78% and 6.48%, respectively.

In Table 5, we present the results achieved by the three-class SVM classification model, in addition to the results for the four-class model. These results were obtained by adopting the SST evaluation strategy, and only case 2 was considered (i.e., TIFs and PC). The average test classification accuracy across subjects was equal to 65.27% in the RH, LH, and F case and 66.17% in the RH, LH, and T case.

A detailed performance comparison between the proposed framework and several existing SOA methods involving multiclass frameworks, using data from the BCI Competition IV dataset IIa, was also conducted, as summarized in Table 6. Besides the well-known methods used by the winners of the competition (the first three rows of Table 6), the most recent channel-based methods were provided by Miao et al., who proposed a feature extraction method for optimizing spatial/frequency/temporal patterns in a data-driven manner [53]. Gaur et al. presented a filtering method based on multivariate empirical-mode decomposition and Riemannian geometry [54]. Finally, Ghanbar et al. proposed a correlation-based common spatial pattern (CCSP), an extension of the traditional CSP method, for both binary and multiclass scenarios [55]. When considering connectivity-based methods, our CST strategy outperformed the methods of both Rodrigues et al. [39] and Uribe et al. [23], meaning that using only BCFs is not sufficient to discern four MI tasks. We present the results in terms of the Cohen’s kappa value for case 2 only (i.e., TIFs and PC). It is important to note that all SOA results refer to the test Cohen’s kappa value achieved by the SST evaluation strategy. With the aim of providing a complete assessment, we report our results for both the SST and CST strategies. Across subjects, the average Cohen’s kappa value was 0.41 using SST and 0.62 using the CST approach. Specifically, the subjects 1, 3, 8, and 9 achieved higher values than the other subjects. Comparing the SOA methods with the results obtained using the CST strategy, we achieved competitive or higher Cohen’s kappa values.

**Figure 4:**
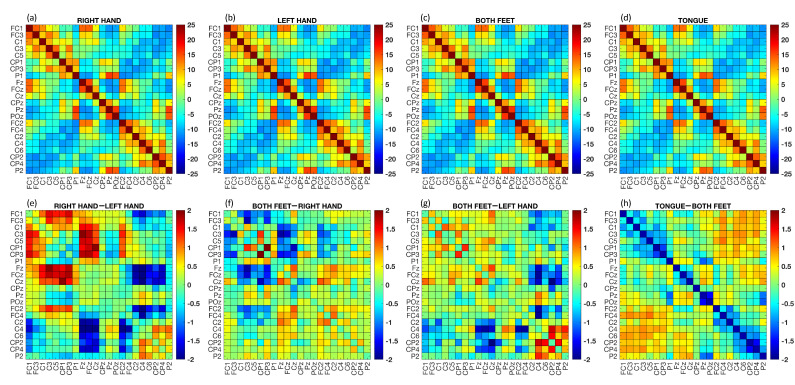
Functional connectivity matrices calculated using the PC method. The Z−score was computed for each matrix associated with a single epoch, and then the average across epochs was calculated. Upper part: grand average PC across subjects and epochs for each MI task ((**a**) RH, (**b**) LH, (**c**) F, and (**d**) T). Lower part: difference between the average PC across subjects and epochs for specific MI tasks ((**e**) RH–LH, (**f**) F–RH, (**g**) F–LH, and (**h**) T–F).

The brain connectivity matrices obtain through the PC metric are illustrated in Figure 4. The upper part of the figure displays the grand average of the FC matrices across subjects and epochs for each class, while the lower part shows the differences between the grand average of some MI tasks. In the lower part of the figure, red points indicate the strongest connections from the first term of the difference, while blue points indicate the strongest connections from the second term of the difference. Visually capturing differences between connectivity matrices can be challenging, but this is often normal due to subtle variations that primarily involve a limited number of connections. By analyzing the difference between RH and LH tasks (Figure 4e), it can be seen that the connections were stronger for channel $C_{3}$ and those closest to it (i.e., $C_{1}$ and $C_{5}$). On the other hand, the PC was weaker for channels $C_{2}$, $C_{4}$, and $C_{6}$. Similar observations can be made by visually inspecting the difference between the F and RH tasks (Figure 4f), where the lowest FC connections involved channels $C_{2}$, $C_{4}$, and $C_{6}$. When comparing the connections between F and T, it can be seen that the highest values were achieved by connections $FC_{2}$ and $FC_{4}$.

## 4. Discussion

In this work, we proposed a novel approach for extracting and merging features based on brain connectivity and an SCN for MI-BCI applications. Despite the growing interest in brain connectivity estimation, the performance in terms of usability and accuracy is still limited, especially when dealing with low-density EEG systems and multiclass problems [6,22]. To overcome this limitation, our approach integrated TIFs with BCFs through a feature fusion approach. We used the SCN to extract channel-based features, which ensured the translation-invariance of the extracted features up to a predefined scale (i.e., invariance scale) [28]. The SCN offered several advantages, including reduced computational complexity, fixed filter weights, interpretable feature extraction, and minimal parameter requirements [26]. Then, different FC metrics, i.e., PC, Pearson correlation, and PLI, were used to account for the brain network functionality.

This combination offered a more complete view of the brain activity, yielding interpretable features and capturing interconnections among brain regions. While traditional methods often rely on single or limited EEG channels, our framework used the power of brain connectivity to overcome limitations in spatial resolution, particularly in scenarios involving spatially approximate brain areas. Moreover, instead of using well-known feature extraction methods in the spatial, frequency, and/or time domain, the employment of the SCN combined with brain connectivity allowed us to improve multiclass MI-BCI resolution by distinguishing closely related classes in terms of spatial organization.

The feature vector was constructed by adopting a feature fusion strategy, followed by the reduction of its dimensionality through the Fisher’s score [24,43]. Finally, a multiclass SVM classifier was used, which is well-accepted in the BCI community for its ability to achieve a high classification accuracy with a relatively low computational complexity [22]. The performance was evaluated in terms of accuracy, Cohen’s kappa value, precision, and recall. This work proposed two evaluation strategies: an SST strategy, wherein the first session of EEG acquisition was used as the training set and the second session as the testing set, and a CST strategy, introduced to overcome the limitations of intra-subject variability among different sessions.

The results shown in Table 1, Table 2 and Table 3 demonstrate that merging TIFs with BCFs improved the discrimination power among different MI tasks. In the SST evaluation approach, case 2 outperformed case 1, with average test accuracies across subjects of 56.10% and 47.82%, respectively (see Table 1 and Table 2). This result was also confirmed by calculating the average test precision and recall. In case 1, the average test precision was 0.47, while in case 2 it was 0.57. The average test recall was 0.48 in case 1 and 0.56 in case 2. Among the different BCFs metrics, TIFs combined with PC performed better; thus, PC was considered for subsequent analysis with BCFs (see Table 3). The average test classification accuracy was 56.10%, 51.31%, and 49.05% when employing PC, Pearson correlation, and the PLI, respectively. These results highlight the importance of considering BCFs to enhance the performance of an MI-BCI pipeline, rather than solely relying on channel-based information.

In the CST evaluation approach, the average test accuracy across subjects was equal to 71.67% in case 2 and 63.25% in case 1 (see Table 4). The results obtained by the CST strategy looked very promising, especially in light of the fact that the average test classification accuracy was 71.67%, higher than the SST results. In addition, it could be observed that in the CST approach the average validation and test classification accuracies were comparable, with a difference of only 6.48% in case 2. These results provide clear evidence of the improvement in the generalization properties of the classifier, highlighting the importance of accounting for data heterogeneity among different EEG sessions. Thus, our second evaluation strategy (i.e., CST) not only yielded a higher performance, but also ensured a more consistent system with a lower SD, combatting the overfitting problem.

In the real-world use of MI-BCI systems, it is well-known that the *calibration* session is time-consuming. The *calibration* phase could be minimized by relying on a recording at rest, when the subjects are not performing any tasks. Using a resting-state measurement can help to address the intra- and inter-subject variability or be used for transfer learning techniques [41,42,56,57]. Unfortunately, the BCI Competition IV dataset IIa does not provide a sufficiently long resting-state signal (of at least a few minutes) [46]. For instance, Gonuguntla et al. treated the time interval between 0.5 and 2 s as rest in a binary classification problem [58] (see Figure 2b). We also explored the option of using the fixation part as rest (see Figure 2b), but we noticed that this portion of the recording could be influenced by the previous trial or by the preparation of the current one. It is worth noting that the patterns of FC are present even during a task-free EEG signal; thus, normalizing the FC matrices could be appropriate (e.g., by computing ERD/ERS). To overcome these real-world limitations, it may be useful to increase the number of EEG acquisitions in order to consider the heterogeneity of data. This would allow one to test the robustness of the MI-BCI system under different conditions and over several days.

Additionally, we computed the grand average of the PC across subjects and epochs for each MI task (see Figure 4). By considering each class separately, the differences in neural connections were not easily discernible, since it is well-known that the four MI tasks involve adjacent brain areas. Nonetheless, by calculating the differences between the RH and LH movements, the strongest patterns were clearly visible in both the right and left central brain areas. These results were consistent with a priori neurophysiological knowledge [30,32]. Moreover, our results showed stronger neural connections in frontal areas for the T task compared to the other tasks [59].

One limitation of this work could be the low spatial resolution of EEG signals acquired during the BCI Competition IV (dataset IIa) [46]. Some brain areas involved in the MI tasks were not properly covered (see Figure 2a). For instance, the imagination of tongue movement is typically mapped bilaterally in the temporal and frontal-temporal brain areas [2,60]; thus, the information coming from electrodes in these areas may have been missing in our model.

To further validate the performance of our method, we conducted a comparison between the proposed framework (i.e., TIFs + PC) with SOA four-class results. Table 6 shows the Cohen’s kappa values for both channel-based and connectivity-based methods achieved by our proposed framework and other SOA approaches, which all referred to an SST evaluation strategy. The average Cohen’s kappa value across subjects achieved by our proposed SST was lower than that of the other SOA methods, except for the method of Rodrigues et al. [39] and the 3rd winner of Competition IV. However, when comparing our results to only connectivity-based SOA results, subjects 1 and 9 outperformed the Cohen’s kappa values of both Rodrigues et al. [39] and Uribe et al. [23]. Rodrigues et al. [39] in 2019 compared the Pearson correlation, Spearman correlation, and mean phase coherence with recurrence-based alternative estimates of brain connectivity. The latter approach outperformed the other measures, but the overall results in terms of performance were still limited. Additionally, Uribe et al. [23] in 2019 defined a framework based on correntropy, degree centrality, and extreme learning machines (ELMs). Although the classification results did not outperform SOA results, they confirmed that brain connectivity should be explored in MI-BCI studies. On the other hand, the average Cohen’s kappa value across subjects achieved by our proposed CST was 0.62, outperforming all the other SOA methods. Observing the Cohen’s kappa values across the two evaluation strategies, we discerned a noteworthy variation between them, as reported for the classification accuracy results in Table 1, Table 2, and Table 4. Thus, the CST method is essential for addressing intra-subject variability across distinct EEG sessions, stemming from temporal shifts in neural processing, the non-stationary nature of EEG signals, and diverse neurophysiological mechanisms. The CST strategy could also be useful to avoid the overfitting problem.

The benefit of the feature fusion approach was also demonstrated by Ai et al. using the same dataset from the BCI Competition IV [44]. In this work, brain functional network features were combined with CSP and LCD. However, a direct comparison with our results was not possible, since we did not find sufficient details in the above work, such as information on CV, training and testing data separation, and whether they reported validation or test results. This issue was also encountered when considering other studies; thus, these works were excluded from the comparison in Table 6 (excluded references: [40,48,61,62]).

## 5. Conclusions

In conclusion, the integration of brain connectivity information into an MI-BCI signal processing pipeline, along with channel-based information, improved the accuracy and Cohen’s kappa values in a multiclass scenario. Our results suggest that FC should be considered instead of relying on information from only one or a few localized brain regions (e.g., channel-based features). The lack of resting EEG data was a limitation of this study. To overcome this issue and capture the intra- and inter-subject variability, we recommend acquiring EEG signals at rest. Furthermore, future work should explore the integration of transfer learning approaches to minimize the *calibration* phase and further improve the control of MI-BCI systems [56,63].

## Figures and Tables

**Table 1:** SST approach. Validation and test evaluation metrics (i.e., accuracy (%), Cohen’s kappa, precision, and recall) achieved by the four-class classifier using only TIFs.

Case 1: TIFs								
**Subject**	**Acc Val.**	**Acc Test**	**k Val.**	**k Test**	**Prec Val.**	**Prec Test**	**Rec Val.**	**Rec Test**
1	93.03	68.33	0.91	0.58	0.94	0.7	0.93	0.68
2	81.11	32.86	0.75	0.11	0.85	0.3	0.81	0.33
3	90.37	52.38	0.87	0.36	0.91	0.52	0.9	0.52
4	85.84	50.44	0.81	0.33	0.89	0.44	0.86	0.5
5	60.56	30.43	0.47	0.07	0.63	0.31	0.61	0.3
6	83.96	40.47	0.79	0.21	0.86	0.39	0.84	0.4
7	84.91	33.21	0.8	0.12	0.87	0.34	0.85	0.33
8	78.72	45.76	0.72	0.28	0.83	0.49	0.79	0.46
9	95.31	76.52	0.94	0.69	0.96	0.79	0.95	0.77
Average	85.76	47.82	0.78	0.30	0.86	0.47	0.84	0.48
SD	10.24	16.08	0.14	0.21	0.10	0.17	0.10	0.16

**Table 2:** SST approach. Validation and test evaluation metrics (i.e., accuracy (%), Cohen’s kappa, precision, and recall) achieved by the four-class classifier using TIFs fused with PC.

Case 2: TIFs + PC								
**Subject**	**Acc Val.**	**Acc Test**	**k Val.**	**k Test**	**Prec Val.**	**Prec Test**	**Rec Val.**	**Rec Test**
1	92.66	79.72	0.90	0.73	0.93	0.80	0.93	0.80
2	74.81	38.16	0.66	0.18	0.75	0.38	0.77	0.35
3	90.37	69.23	0.87	0.59	0.92	0.70	0.90	0.69
4	86.61	56.14	0.82	0.41	0.89	0.52	0.87	0.56
5	60.57	37.32	0.47	0.16	0.6	0.37	0.61	0.37
6	86.73	41.86	0.82	0.22	0.89	0.44	0.87	0.42
7	86.03	35.02	0.81	0.14	0.88	0.43	0.86	0.35
8	90.85	67.53	0.88	0.57	0.93	0.70	0.91	0.68
9	95.27	79.92	0.94	0.73	0.96	0.83	0.95	0.80
Average	84.88	56.10	0.80	0.41	0.86	0.57	0.85	0.56
SD	10.81	18.54	0.15	0.25	0.11	0.18	0.10	0.19

**Table 3:** SST approach. Comparison of BCFs obtained with phase correlation (PC), Pearson correlation, and the phase lag index (PLI). Validation and test accuracy (%) achieved by the four-class classifier are reported.

Case 2	TIFs		TIFs		TIFs	
	**PC**		**Pearson Correlation**		**PLI**	
**Subject**	**Validation**	**Test**	**Validation**	**Test**	**Validation**	**Test**
1	92.66	79.72	91.64	71.17	94.14	72.24
2	74.81	38.16	52.97	33.57	51.89	33.21
3	90.37	69.23	76.67	64.10	80.37	59.34
4	86.61	56.14	72.45	46.49	62.19	45.17
5	60.57	37.32	61.61	35.87	63.56	34.78
6	86.73	41.86	72.55	38.14	70.26	37.67
7	86.03	35.02	72.33	34.66	74.15	36.46
8	90.85	67.53	87.11	59.41	82.55	48.34
9	95.27	79.92	95.72	78.41	93.62	74.24
Average	84.88	56.10	75.89	51.31	74.75	49.05
SD	10.81	18.54	13.84	17.27	14.36	15.95

**Table 4:** CST approach. Validation and test accuracy (%) achieved by the four-class classifier in case 1 (i.e., TIFs) and case 2 (i.e., TIFs + PC).

	Case 1: TIFs		Case 2: TIFs + PC	
**Subject**	**Validation**	**Test**	**Validation**	**Test**
1	86.47	70.41	88.79	83.43
2	57.61	52.63	62.29	56.14
3	79.55	72.05	82.18	88.82
4	77.50	60.34	81.00	60.34
5	67.35	59.15	56.89	57.93
6	71.90	57.28	76.16	68.93
7	75.50	51.52	74.66	60.61
8	73.88	61.64	86.91	77.36
9	93.96	84.21	94.50	91.45
Average	75.99	63.25	78.15	71.67
SD	10.50	10.50	12.25	13.89

**Table 5:** SST approach. Validation and test accuracy (%) achieved by the three-class classifier—case 2: TIFs + PC.

	RH, LH, and F		RH, LH, and T	
**Subject**	**Validation**	**Test**	**Validation**	**Test**
1	92.14	89.52	92.57	91.04
2	70.26	49.29	78.40	43.46
3	92.62	75.12	92.64	90.24
4	85.05	78.86	90.74	57.40
5	72.11	40.10	82.71	44.12
6	64.41	43.21	90.00	50.31
7	95.00	54.98	94.12	47.57
8	73.76	71.92	92.50	82.18
9	96.01	84.42	100	89.23
Average	82.37	65.27	90.40	66.17
SD	12.26	18.58	6.36	21.39

**Table 6:** Proposed framework (i.e., TIFs + PC) compared with other SOA methods in terms of Cohen’s kappa value for each subject (BCI Competition IV dataset IIa).

Method	1	2	3	4	5	6	7	8	9	Avg.
Channel-based methods										
Ang et al., 2012 (1st winner)	0.68	0.42	0.75	0.48	0.40	0.27	0.77	0.75	0.61	0.57
Guangquan et al., 2008 (2nd winner)	0.69	0.34	0.71	0.44	0.16	0.21	0.66	0.73	0.69	0.52
Song, 2008 (3rd winner)	0.38	0.18	0.48	0.33	0.07	0.14	0.29	0.49	0.44	0.31
Gouy-Pialler et al., 2009 [52]	0.66	0.42	0.77	0.51	0.50	0.21	0.30	0.69	0.46	0.50
Wang et al., 2012 [35]	0.56	0.41	0.43	0.41	0.68	0.48	0.80	0.72	0.63	0.57
Miao et al., 2017 [53]	0.63	0.43	0.74	0.54	0.19	0.26	0.63	0.62	0.69	0.53
Gaur et al., 2018 [54]	0.86	0.24	0.7	0.68	0.36	0.34	0.66	0.75	0.82	0.60
Ghanbar et al., 2021 [55]	0.72	0.40	0.70	0.55	0.20	0.35	0.66	0.78	0.77	0.57
Connectivity-based methods										
Rodrigues et al., 2019 [39]	0.46	0.13	0.56	0.26	0.11	0.11	0.16	0.60	0.56	0.33
Uribe et al., 2019 [23]	0.66	0.27	0.69	0.36	0.23	0.32	0.46	0.66	0.64	0.48
Proposed TIFs + PC										
*SST strategy*	0.73	0.18	0.59	0.41	0.16	0.22	0.14	0.57	0.73	0.41
*CST strategy*	0.78	0.42	0.85	0.47	0.44	0.59	0.48	0.70	0.89	0.62

## Data Availability

A publicly available dataset was analyzed in this study. These data can be found at: https://www.bbci.de/competition/iv/ accessed on 7 November 2022.

## Abbreviations

The following abbreviations are used in this manuscript:

MI-BCI	Motor-imagery brain–computer interface
EEG	Electroencephalography
ERD	Event-related desynchronization
ERS	Event-related synchronization
EOG	Electrooculography
FC	Functional connectivity
EC	Effective connectivity
PC	Phase correlation
PLI	Phase lag index
wPLI	Weighted phase lag index
PDC	Partial directed coherence
PLV	Phase lag value
CSP	Common spatial pattern
LCD	Local characteristic decomposition
CCSP	Correlation-based common spatial pattern
OVR-FBCSP	One-versus-the-rest filter-bank common spatial pattern
TIFs	Translation-invariant features
SCN	Scattering convolution network
BCFs	Brain connectivity features
SVM	Support vector machine
LDA	Linear discriminant analysis
k-NN	k-nearest neighbor
CNN	Convolutional neural network
RNN	Recurrent neural network
RBF	Radial basis function
OVR	One-versus-the-rest
SST	Session-to-session transfer
CST	Calibration-session transfer
CV	Cross-validation
SD	Standard deviation
LH	Left hand
RH	Right hand
F	Feet
T	Tongue
ML	Machine learning
SOA	State-of-the-art
ELMs	Extreme learning machines
